# Female employees' perceptions of organisational support for breastfeeding at work: findings from an Australian health service workplace

**DOI:** 10.1186/1746-4358-6-19

**Published:** 2011-11-30

**Authors:** Danielle Weber, Anneka Janson, Michelle Nolan, Li Ming Wen, Chris Rissel

**Affiliations:** 1 Health Promotion Service, Hugh Jardine Building, Eastern Campus Liverpool Hospital, Liverpool, New South Wales, Australia

**Keywords:** Breastfeeding, workplace, organisational support

## Abstract

**Background:**

Women's return to work can be a significant barrier to continued breastfeeding. Workplace policies and practices to promote and support continued, and longer duration of, breastfeeding are important. In the context of the introduction of a new breastfeeding policy for Area Health Services in New South Wales, Australia, a baseline survey was conducted to describe current practices and examine women's reports of perceived organisational support on breastfeeding intention and practice.

**Methods:**

A cross sectional survey of female employees of the Sydney South West Area Health Service was conducted in late 2009. A mailed questionnaire was sent to 998 eligible participants who had taken maternity leave over the 20-month period from January 2008 to August 2009. The questionnaire collected items assessing breastfeeding intentions, awareness of workplace policies, and the level of organisational and social support available. For those women who had returned to work, further questions were asked to assess the perceptions and practices of breastfeeding in the work environment, as well as barriers and enabling factors to combining breastfeeding and work.

**Results:**

Returning to work was one of the main reasons women ceased breastfeeding, with 60 percent of women intending to breastfeed when they returned to work, but only 40 percent doing so. Support to combine breastfeeding and work came mainly from family and partners (74% and 83% respectively), with little perceived support from the organisation (13%) and human resources (6%). Most women (92%) had received no information from their managers about their breastfeeding options upon their return to work, and few had access to a room specially designated for breastfeeding (19%). Flexible work options and lactation breaks, as well as access to a private room, were identified as the main factors that facilitate breastfeeding at work.

**Conclusions:**

Enabling women to continue breastfeeding at work has benefits for the infant, employee and organisation. However, this baseline study of health employees revealed that women felt largely unsupported by managers and their organisation to continue breastfeeding at work.

## Background

Breastfeeding is one of the most natural, protective and cost effective practices a mother engages in with her new infant [1-3]. Australian breastfeeding initiation rates at both the national and state level are high (around 90 percent) [4-6], but by three months, exclusive breastfeeding has dropped to 50 to 60 percent, and is at 15 percent at six months, well below the recommendation of exclusive breastfeeding for the first six months of life [4-9]. Breastfeeding continues to be major public health priority at both state and national levels [9,10].

With women now representing 46 percent of the Australian labour force, it is evident that women make a significant contribution to the national economy [11]. Twenty one percent of previously working Australian women resume employment in the first six months following childbirth, and 42 percent by 12 months [4]. A woman's return to work has repeatedly been found to be a major contributor to the premature cessation of breastfeeding [12-19].

For the employer, the benefits of providing a working environment conducive to breastfeeding outweigh the costs. If breastfeeding is supported in the workplace, women are more likely to return to work, and return earlier, which contributes to women maintaining their job skills, as well as reducing staff turnover [20-22]. Women are also more likely to have reduced frequency and length of work absenteeism due to fewer and less severe baby-related illnesses [20-22]. Additionally, women are more likely to have higher morale and improved levels of concentration, resulting in increased productivity [20-22]. Accommodating breastfeeding mothers may also contribute towards the development of a positive corporate image [21,23].

Workplaces can be an ideal setting for implementing policies and practices to promote and support the continuation and longer duration of breastfeeding [15,21]. Studies indicate that mothers who have convenient access to their infant during the work day, or who expressed breast milk at work, have longer breastfeeding duration than other mothers [24,25].

Support from staff and management, access to facilities to feed their infant or express and store breast milk, flexible hours and lactation breaks have all been identified as crucial elements required by women to successfully continue breastfeeding on their return to work [4,15,17,20,26-31]. A study by Dodgson, Chee and Yap [32] examining workplace breastfeeding policies and practices in Hong Kong hospitals found those hospitals with a committee addressing workplace breastfeeding issues achieved a more supportive environment than those hospitals without. Furthermore, development of a strategic plan and a positive attitude towards breastfeeding at work significantly enhanced the likelihood of policy success [29,33].

The aim of this study was to examine the attitudes, breastfeeding practices and experiences of female employees in an Australian health service workplace who had returned to work from maternity leave, and to investigate their perceived level of organisational support to combine breastfeeding and work. This baseline survey forms a component of an evaluation, using a before and after study design, of a workplace intervention to encourage and support health service employees to combine breastfeeding and work.

The Sydney South West Area Health Service (SSWAHS), Australia looks after all public hospitals and health care facilities (eg early childhood clinics, community health centres) in the central and south-west regions of Sydney, New South Wales (NSW). It covers an area of 6380 square kilometres and contains 35 health facilities, including 11 hospitals. The workforce is predominately female (74%) and in 2007 numbered 23 419 employees [34]. Staff are employed in a diverse range of clinical and non-clinical occupations, including doctors, nurses, allied health, population health, administration, management, information technology, and domestic services. Clinical staff may have less flexibility in their daily workload due to patient demands, which could impact on their ability to take regular lactation breaks.

At the time of the survey, full time and permanent part time health service employees who had completed 40 weeks continuous service were entitled to 14 weeks paid maternity leave and a further period of unpaid maternity leave of up to 12 months from the date of birth of their child. With management agreement, employees could also return from maternity leave on a part time basis until their child reached school age. Staff employed under the Nursing and Midwifery Award were entitled to a 30 minute paid lactation break per eight hour shift. Staff employed under other awards who wished to breastfeed or express breast milk during work time had to do so during their allocated meal breaks or unpaid time. Of the 35 SSWAHS facilities, only two had a dedicated staff breastfeeding room.

## Methods

### Study design

A cross sectional survey using a mailed questionnaire was conducted in central and south-west Sydney, Australia, during November and December 2009.

### Study participants

A convenience sample of 998 female employees from SSWAHS who had valid home addresses and had taken maternity leave over the 20 month period from January 2008 to August 2009 were eligible to participate. Eligible participants were identified (including de-identified home addresses) by the SSWAHS Human Resources database. Forty questionnaires were returned due to incorrect addresses and subtracted from the denominator. Completion of the questionnaire was taken as evidence of consent to participate.

### Data collection and key measures

A self-complete 59-item questionnaire, including a return prepaid envelope, was mailed to eligible participants in November and December 2009. Two reminder letters were sent to non-respondents, the first approximately three weeks after the initial mail-out and again three weeks later.

The survey comprised of three sections, with participants asked to complete each subsequent section of the survey based on their responses to certain categorical questions.

Section 1 consisted of 27 items which all participants were required to complete. In this section, women were asked their age, education level, household income, language spoken at home and employment status using standard questions from the NSW Health Survey [35]. Women were also asked to recall details relating to their most recent maternity leave including parity, length of maternity leave, intention to breastfeed prior to baby's birth, intention to return to work, their awareness of organisational policies or facilities to support breastfeeding at work, and whether they received any information from their employer about options to continue to breastfeed when they returned to work. Women were also asked about their method of infant feeding and whether they returned to work at SSWAHS following maternity leave.

Women who indicated they had returned to work were asked fixed item questions regarding the age of their child when they returned to work, hours worked per week, child care arrangements and open-ended questions on the benefits and difficulties of returning to work.

In section 2, only women who indicated they had breastfed at birth (whether they returned to work or not), were asked 7 fixed-item questions about their breastfeeding practices including duration, intention to breastfeed when returning to work, whether they communicated with management about their breastfeeding and work requirements prior to returning to work, and whether receiving information about organisational support available to combine breastfeeding and work would have influenced their decision to do so. Where relevant, women were also asked two open-ended questions related to reasons for stopping breastfeeding, and reasons for not continuing to breastfeed after returning to work.

In section 3, women who indicated they continued to breastfeed when they returned to work were asked a further 18 fixed-choice questions assessing their perceptions and practices of breastfeeding in the work environment including availability and features of facilities, lactation breaks, and perceived level of organisational and social support. Women were asked to select relevant enablers and barriers to breastfeeding and work from a pre-determined list.

### Ethics

The study was approved by the Ethics Review Committee of Sydney South West Area Health Service (Royal Prince Alfred Hospital Zone), Protocol No X09-0216 and HREC/09/RPAH/353.

### Analysis

Statistical analyses were carried out using SPSS (Version 15). Relationships between study and outcome variables were examined using a Pearson chi-square test.

## Results

Of a total of 998 surveyed, 496 women completed the survey representing a response rate of 51.7 percent. A full report of the results is available from the corresponding author.

### Characteristics of respondents

The mean age of women completing the survey was 35 years and the majority were married, of non-Aboriginal descent, and university educated (84%) (Table 1). Just over a third of women spoke a language other than English (LOTE) at home, which is representative of the local area. The average household income for more than half of the survey participants was high, being A$80,000 (Table 1). Occupation responses were coded into three categories "nurse/midwife", "administration/management" and "clinical/allied health". Almost half (49%) of respondents were a nurse or midwife, 39 percent were in a clinical or allied health role and a further 12 percent in administrative or management roles (Table 1).

**Table 1:** Characteristics of survey participants employed by the Area Health Service (n = 496)

Population characteristic	N	%
**Maternal indigenous status ^Ω^**		
Aboriginal	2	1
Non-Aboriginal	304	99
**Maternal Background ^Ω^**		
English speaking	323	66
Non-English speaking	168	34
**Relationship status ^Ω^**		
Married/De-facto	480	97
Divorced/Separated	7	1
Never married	8	2
**Maternal Education ^Ω^**		
University*	414	84
TAFE#	63	13
Less than tertiary¥	15	3
Other	3	1
**Current maternal employment status**		
Full time employment¤	92	19
Part time employment	161	33
Maternity leave	220	44
Other€	23	5
**Occupation**		
Nurse/midwife	242	49
Clinical/Allied Health	190	39
Administration/Management	61	12
**Approximate household income (before tax); last 12 months (in Australian dollars) ^Ω^**		
Less than A$39999	48	10
A$40000-A$79999	157	33
A$80000 or more	278	58
**Age of infant when returned to work ^Ω^**		
Less than 6 months old	41	11
6 months to less than 12 months old	192	54
12 months old or more	123	35

* Including other tertiary institute degree or higher.

# Technical or trade certificate or diploma, ¥ School certificate or higher school certificate.

^Ω ^Not all women answered the question, therefore total is less than 496

¤Includes full time, part time and casual. € Includes un-employed, home duties and any others.

### Awareness of workplace breastfeeding policies and facilities

At the time of the survey, women were entitled to maternity leave, flexible work practices and, for women employed under the nursing and midwifery award, a paid 30 minute lactation break per shift. Most respondents were aware of their maternity leave entitlements (90%; n = 446) and to a lesser extent the option of flexible work practices (63%; n = 312). At least half of the midwives responding to the survey were aware of their lactation break entitlement (n = 38) but only 28 percent of general nurses were aware of this entitlement (n = 204). Amongst all women surveyed, awareness of a breastfeeding room was low (14%) reflecting the fact that only two area health facilities had a designated room available at the time.

Ninety seven percent of women indicated that prior to their baby's birth they intended to breastfeed, with 98 percent of women reporting that their infant was "ever breastfed" (n = 419). Breastfeeding rates declined in the first few months, with 13 percent (n = 29) stopping breastfeeding by 12 weeks (3 months), and a further 24 percent (n = 54) by 24 weeks (6 months).

Only five percent of the 493 respondents had been given written or verbal information from their employer about the option to continue breastfeeding upon their return to work. Almost all mothers (95%; n = 470) intended to return to work following maternity leave, with another four percent undecided. At the time of the survey, 52 percent of mothers were working full or part time (n = 253), while 44 percent were still on maternity leave (n = 220). For those women whose maternity leave had finished, 79 percent (n = 327) stated they returned to work at SSWAHS following their maternity leave, indicating a high staff retention rate among the organisation. Most women (86%, n = 300) returned to work part time (less than 35 hours per week), with two thirds returning before their baby's first birthday (less than 6 months of age, 11.1%; 6-11 months of age, 54%).

### Breastfeeding intention and practice upon return to work

Returning to work was the second most frequent response to the question "What was the main reason for stopping breastfeeding your child?" Of the 259 women who had reportedly ceased breastfeeding at the time of the survey, 25 percent reported returning to work as the main reason why they stopped breastfeeding (n = 65). Other main reasons included: that the mother felt the infant was ready to stop (31%, n = 80) and that milk supply was insufficient (19%, n = 50).

Of 390 respondents, nearly 60 percent of women (n = 230) had considered the possibility of breastfeeding after returning to work, but only 40 percent (n = 156) continued to do so upon return to work (Table 2). For those women who had considered breastfeeding on return to work but had not, the main reason given was a lack of breastfeeding/expressing facilities and a lack of workplace and managerial support (44%, n = 66). Other reasons included the infant was ready to stop (19%, n = 29) and that women felt it was too difficult or inconvenient (19%. n = 28). Eight women (5%) had planned to stop breastfeeding upon their return to work.

**Table 2:** Continuation of breastfeeding on a mothers return to work

Characteristic	N	%
**Considered combining breastfeeding & work**		
Yes	230	59
No	160	41
**Did combine breastfeeding & work**		
Yes	156	40
No	235	60
**How combined breastfeeding & work**		
Breastfed before & after work/infant formula during work hours	57	37
Breastfed before & after work/expressed breast milk during work hours	56	36
Breastfed baby before/after/during work hours	2	1
Other	40	26

Education level, occupation and income had no effect on intention and practice of breastfeeding, nor did the age of the infant or part-time status when the mother returned to work.

Only eight percent of women (n = 29) had spoken to their manager about breastfeeding prior to returning to work, although nearly 60 percent felt that they "would have been more likely to continue to breastfeed after returning to work" if they had received information and support about this possibility prior to going on maternity leave (n = 205).

### Breastfeeding practices at work

When asked "how did you continue to breastfeed after you returned to work?" 36 percent reported combining breastfeeding and expressing milk at work, while only two women (1%) were able to breastfeed their baby during work hours. More than a third of women did not breastfeed or express milk at work, preferring to breastfeed before and after work, and have the infant provided with infant formula while they were away (Table 2).

When women were asked "When did you breastfeed or express while at work?" the most common response was during allocated meal break times (57%), followed by additional paid lactation breaks (16%), and additional unpaid lactation breaks/own time (14%). Forty two percent (n = 34) found their breaks for breastfeeding or expressing to be inflexible.

Most breastfeeding women used a manual pump (51%) or electric pump (33%) to express, and most used their own pump (83%). Expressed milk was usually stored in the staff refrigerator. Only 20 percent (n = 14) of women expressed or breastfed in a room especially designated for the purpose, with one in four women using their office or another location (54%).

When questioned about the qualities of the room where they breastfed, most women reported it to be clean (87%), within a five minute walk of their work station (90%) and providing access to a power point (83%) and cleaning facilities (79%). However, two thirds stated that the room was not always available when needed and only 57 percent reported it to be private. A comfortable chair was reported to be available in just over half of rooms.

### Breastfeeding support at work

Women reported several factors that enabled them to continue breastfeeding whilst at work. Flexible work options (including working part time or reduced hours) (17%, n = 71) and flexibility of break times (11%, n = 40) were the most commonly mentioned enablers. Support from management and colleagues (11% (n = 43) and 13% (n = 51) respectively) were also important, as was access to a private room for breastfeeding or expressing (n = 40). Similar responses were given when women were asked to nominate factors that made it difficult to breastfeed at work. Nearly 20 percent stated inflexible break times and a lack of lactation breaks made breastfeeding difficult (n = 77). Role overload due to multiple demands (n = 60), lack of access to a private room (n = 49) and lack of available information (n = 39) were also issues reported.

Support to combine breastfeeding and work came mainly from family and partners. Many women listed the organisation (61%, n = 71) and human resources department (70%, n = 81) as providing no support. Line managers and co-workers offered a varied level of support (Table 3).

**Table 3:** Perceived level of support felt by women combining breastfeeding and work

Source of support	Level of support						
	
	Nil		Moderate		High		
	
	N	%	N	%	N	%	Total ^Ω^
Organisation	71	61	31	27	15	13	117
Line manager	42	35	44	37	34	28	120
Human Resources	81	70	27	24	7	6	115
Colleagues/co-workers	31	25	50	41	41	34	122
Family	6	5	28	22	96	74	130
Partner	4	3	19	14	109	83	132

^Ω ^Not all women were eligible to answer the question, therefore total is less than 496

Workplace characteristics and intention to, and practice of, breastfeeding at work were examined. Receiving either verbal or written information about breastfeeding was not significantly associated with women's intention to breastfeed or breastfeeding practice (p = 0.29 and p = 0.89 respectively), nor was a high level of support from partner/family (p = 0.97 and 0.68 respectively) or organisation (p = 0.80 and p = 0.66). However, small cell sizes limit the meaningfulness of these analyses.

When questioned about their experience of breastfeeding at work, 58 percent found it to be very positive or positive, 23 percent were neutral and 21 percent found it to be a negative or very negative experience. Women were asked to provide a reason for their response regarding their breastfeeding experiences, and comments from the few women who responded included:

"*It was stressful worrying about needing to express regularly, especially when work was busy. I had to cover a shared office window with a pillowslip for privacy and still was nervous someone would walk in on me*."

"*Flexible hours made it easier to combine the two. Very supportive boss and coworkers. Worked from home and attended work only as required"*

The only suitable place was a toilet (as it had privacy) but who wants to sit on a toilet for 20 minutes to express?"

## Discussion

This baseline study of women employed by an Australian area health service revealed that employees felt largely unsupported by managers and their organisation to continue breastfeeding at work. Support to combine breastfeeding and work came mainly from family and partners with little perceived support from the organisation and human resources. However, there was no statistically significant association between organisational support and intention to, or practice of, breastfeeding at work. The small numbers in subgroup analyses are likely to be a contributing factor to the lack of a clear association, as well as the strong support from family and partners, which may have overshadowed support offered by the organisation.

Returning to work was one of the main reasons women ceased breastfeeding, similar to findings in other studies [5,12,13,15], with 60 percent of women intending to breastfeed, but only 40 percent continuing to do so on return to work. Flexible breaks and work options, as well as access to a private room facilitated breastfeeding at work.

The particular characteristics of this study population, being highly educated women working in an area health service may limit comparability of these results with other workplace studies. A limitation of the study is that no assessment of overall workplace and management support for all staff was made, and it is possible that non-breastfeeding employees may also perceive organisational support to be minimal. The low response rate also limits the generalisability of the study. Ideally, the timeframe between the sample population of women and when the survey was conducted should have been wider to allow the opportunity for more women to respond once their maternity leave was finished and they had returned to work.

Enabling women to continue breastfeeding at work has benefits for the infant, employee and organisation [2,21,22]. However, as evidenced by findings in this study, despite a strong desire to continue breastfeeding, women need greater support in the workplace if they are to successfully combine breastfeeding and work. Organisations need to create a workplace culture that supports and promotes breastfeeding, and institute policies that allow flexible hours, lactation breaks and appropriate facilities to express and store breast milk [17,20,26,29]. This baseline study was conducted prior to the implementation of the SSWAHS breastfeeding policy, and has highlighted areas that require improvement to better support breastfeeding employees. The results of the post policy survey will hopefully reveal greater support for women to breastfeed, resulting in higher rates of sustained breastfeeding among SSWAHS employees.

## Conclusions

The transition period of returning to work is a critical time to support the continuation of breastfeeding amongst female employees. Workplaces and employers have a crucial role in providing supportive workplace environments, appropriate facilities, strong management support, and relevant policies in order for women to feel adequately supported and encouraged to continue to breastfeed when returning to work.

## List of abbreviations

LOTE: Language other than English; NSW: New South Wales; SSWAHS: Sydney South West Area Health Service.

## Declaration of conflicting interests

The authors declare that they have no competing interests.

## Authors' contributions

MN and CR conceived the study and participated in its design. AJ conducted the literature review and analysed the data. DW, LMW and CR interpreted the data and drafted the manuscript. MN revised the manuscript. All authors read and approved the final manuscript.
